# Health care utilization at the end of life in Parkinson’s disease: a population-based register study

**DOI:** 10.1186/s12904-024-01581-6

**Published:** 2024-10-29

**Authors:** Breiffni Leavy, Elisabet Åkesson, Johan Lökk, Torbjörn Schultz, Peter Strang, Erika Franzén

**Affiliations:** 1 Division of Physiotherapy, Department of Neurobiology, Care Science and Society, Karolinska Institutet, Stockholm, Sweden; 2 Research and Development Unit, Stockholm Sjukhem Foundation, Stockholm, Sweden; 3 Division of Neurogeriatrics, Department of Neurobiology, Care Science and Society, Karolinska Institutet, Stockholm, Sweden; 4 Division of Clinical Geriatrics, Department of Neurobiology, Care Science and Society, Karolinska Institutet, Stockholm, Sweden; 5 Department of Geriatrics, Karolinska University Hospital, Stockholm, Sweden; 6 Department of Oncology-Pathology, Karolinska Institutet, Stockholm, Sweden; 7 Medical unit Allied Health care professionals, Theme Women’s Health and Allied Health Professionals, Karolinska University Hospital, Stockholm, Sweden

**Keywords:** End-of-life, Parkinson’s disease, Health care utilization, Specialized palliative care, Nursing homes

## Abstract

**Background:**

Knowledge of health care utilization at the end of life in Parkinson’s disease (PD) is sparse. This study aims to investigate end of life health care utilization, characterized by emergency room (ER) visits, receipt of specialized palliative care (SPC), and acute hospital deaths in a Swedish population-based PD cohort.

**Methods:**

We conducted a retrospective cohort study on deceased patients (≥ 18 years) with a PD diagnosis during their last year of life (*n* = 922), based on health care-provider data from Region Stockholm´s data warehouse, for the study period 2015–2021. Univariable and multivariable logistic regression analyses tested associations and adjusted Odds ratios (aORs) were calculated.

**Results:**

During the last month of life, approx. half of the cohort had emergency room (ER) visits and risk of frailty (measured by Hospital Frailty Risk Score) significantly predicted these visits (aOR, 3.90 (2.75–5.55)). In total, 120 people (13%) received SPC during their last three months of life, which positively associated with risk for frailty, (aOR, 2.65 (1.43–4.94, *p* = 0.002). In total, 284 people (31%) died in acute hospital settings. Among community-dwellers, male gender and frailty were strongly associated with acute hospital deaths (aOR, 1.90 (1.15–3.13, *p* = 0.01) and 3.70 (1.96–6.98, *p* < 0.0001)).

**Conclusions:**

Rates of ER visits at end of life and hospital deaths were relatively high in this population-based cohort. Considering a high disease burden, referral to SPC at end of life was relatively low. Sex-specific disparities in health care utilization are apparent. Identifying people with high risk for frailty could assist the planning of optimal end-of-life care for people with PD.

## Background

People at late-stage Parkinsons disease (PD) present with complex symptoms and high levels of disability [1]. As motor symptoms become less responsive to levodopa [2, 3], the prevalence of non-motor manifestations such as autonomic and sleep dysfunction, musculoskeletal and dyskinesia-related pain, as well as gastrointestinal and ophthalmologic symptoms increases [1]. Additionally, at the late disease stage, cognitive impairment and neuropsychiatric symptoms, characterized by depression and anxiety, frequently prevail alongside apathy, agitation, or psychosis [1, 4, 5].

Clinically relevant milestones have been identified throughout PD disease progression, whereby at the late stage, falls, dysphagia and dementia drastically affect quality of life and place an overwhelming strain on caregivers [6, 7]. When home help and outpatient health care visits become insufficient to manage the plethora of progressive symptoms, this commonly leads to nursing home (NH) placement [8, 9]. Notably, the shift to NH residence in PD has been reported to lead to reduced contact with specialist neurological care [10, 11]. In Sweden, where health care is publicly funded and policies strongly promote ageing in community settings for as long as possible, an older person must demonstrate substantial dependency in activities of daily life to be eligible for NH admission. Subsequently, the average age on admission (85 years), prevalence of cognitive impairment (67%) and ADL dependency (56%) among NH residents is high [12, 13]. For the entire population in 2022, approx. 1.5% of people aged 65–79 years, and 14% of those 80 years and older lived in permanent NH residence [12].

At end of life in PD, the symptom burden is comparable to metastatic cancer [14], and the need for psychosocial and existential support is similar [15]. For these reasons, specialized palliative care (SPC) is often appropriate, yet seldom offered [15]. In Sweden (Stockholm specifically), around-the-clock availability of specialized palliative home care is the basis for SPC. This home care is run by a multiprofessional team, including physicians, nurses, physiotherapists, occupational therapists, dieticians, and social workers. When needed, those admitted to specialized palliative home care can be transferred to specialized inpatient palliative care wards, that are typically run by the same SPC service [16]. This structure appears to be highly appreciated both by patients and their families [17].

Delivering high-quality end-of-life care which meets the complex needs in people with PD, requires planning, whereby a first step is to understand the current health care utilization of this group. Numerous indicators of appropriate end-of life care have been identified in palliative cohorts, and when using population-level health register data, determinants such as emergency room (ER) visits, receipt of SPC and place of death are frequently cited determinants [18, 19]. Existing evidence, sourced primarily in North American PD cohorts indicates that the frequency of acute hospitalizations, and hospitals as places of death is high, and that palliative care is underutilized [11, 20, 21]. Knowledge concerning factors affecting health care utilization is sparse, particularly in relation to health (frailty), sex and socioeconomic-related factors.

## Methods

### Aims

This study aimed to increase knowledge of health care utilization at the end of life in PD, by investigating the frequency of ER visits during the last month of life, access to SPC during the last three months of life, as well as place of death in a population-based patient Swedish cohort. Additionally, we aimed to examine whether health care utilization differed in relation to health (frailty), sex, residential or socioeconomic status. The Strengthening the Reporting of Observational Studies in Epidemiology (STROBE) criteria were used to report Methods and Results [22].

### Study design and setting

This retrospective observational study was based on administrative health care data from Region Stockholm´s data warehouse (VAL) in Sweden[23]. The study period was from 2015 to 2021 and based on data from Stockholm County, which covers about 2.4 million inhabitants. The VAL data registry contains data concerning all health care contacts occurring in all forms of primary care, hospital visits, appointments, in-ward care episodes, as well as diagnoses according to WHO ICD-10 classification. Nationally used KVÅ-codes (*Klassifikation av Vårdåtgärder*, appr. “National classification of care interventions”, performed in outpatient or inpatient care) are also reported to VAL. Reporting to the VAL database is mandatory for all health care workers. The Swedish health care system is funded by taxes and publicly available to all its citizens, i.e., the choice of treatment and medical services is based on medical needs, not on available insurance.

### Study population

All deceased patients 18 years and older with a diagnosis of PD (G20 according to ICD-10 classification) were included, with certain restrictions: As the VAL database does not include death certificates, only main and secondary ICD-10 diagnoses for each appointment or care period, we made the following screening and subsequent selections: 1) First we screened for all patients with an ICD-10 diagnosis of PD, as their main or secondary diagnosis during their last year of life, which gave us 2427 patients. 2) As we aimed to study the care use mainly related to PD, we chose only those with PD as their main diagnosis when receiving health care, during last year of life, which amounted to 1752 patients. 3) As we were mainly interested in the care provided at the end-of-life, we scanned for patients with PD as their main diagnosis during the last three months, which gave us 1065 patients. 4) Finally, we removed those among the 1065 patients who a) were not receiving at least one typical PD medication (*n* = 71) and b) had a concomitant diagnosis of advanced cancer, as this would affect palliative care utilization (*n* = 70), and c) had missing data (missing Mosaic group, *n* = 2), which resulted in 922 patients in the study sample. The study sample comprised of the entire cohort between 2015–2021 and therefore no power calculations were made.

### Variables

As outcome measures, we used receipt of SPC, ER visits, and hospital deaths. As explanatory variables we used age, sex, socio-economic Mosaic groups on area level, risk of frailty as measured by the Hospital Frailty Risk Score (HFRS) [24]. In the analysis of ER visits and hospital deaths, receipt of SPC was used as used as an explanatory variable. Receipt of SPC involves the proportion of patients who were referred, accepted and subsequently received SPC (specialized palliative home care and/ or specialized palliative in-patient care) at the end of life. Mosaic is a commercial socio-economic measure on an area level, to which Stockholm Region subscribes. Using Mosaic, the county is divided into about 1300 small areas, which are labelled as Mosaic 1, 2 or 3, based on socio-economic variables such as income and education, but also on more than 40 additional variables including living arrangements, cultural aspects, and lifestyle. Mosaic group 1 areas are the most affluent ones, whereas Mosaic group 3 comprises less affluent areas. In this analysis, we merged Mosaic group 1 + 2 which we compared with Mosaic group 3.

HFRS is a validated measure of the risk of frailty, based on 109 weighted ICD-10 diagnoses, that have been found to be more prevalent in frail persons [24]. The risk of frailty is categorized as low (< 5 points), intermediate (5–15 points), or high (> 15 points) and in the analyses we compared those with low risk (< 5 points) with intermediate and high risk (5 points or more). The lookback window was one year from the time of death for each of the included patients.

### Statistical analysis

T-tests were used for comparison of means and substituted with Wilcoxon Rank Sum test (Mann–Whitney U test) for comparisons with skew distributions while Chi-square tests were applied for comparison of proportions. Initially, univariable logistic regression analyses were performed. Multivariate logistic regressions were performed to determine the likelihood of ER visits, receipt of SPC and acute hospital deaths. Explanatory variables to be included in the multivariate logistic regression were purposively selected based on clinical relevance and the previous evidence in the published literature, and adjusted Odds ratios (aORs) were calculated [25]. The SAS 9.4/Enterprise guide 8.2 was used for carrying out data analyses. Since the inclusion is based on ICD-10 codes and data is mandated to report and the base of the health care providers’ economic compensation, missing data are very few (estimated to be < 1%).

## Results

### Demographics and clinical data

In total, 922 persons (63% men and 37% women) who had PD as their main diagnosis during the last three months of life and died with PD were included in the analyses, of which 60% were NH residents. The mean age for the entire group was 80.2 years (SD: 7.0): 80.6 years (SD: 6.8) for NH residents and 79.5 (SD: 7.3) years for others (Table 1). Risk of frailty, as measured by HFRS, was seen in 717 (78%) of the studied persons and 28% resided in less advantaged socioeconomic areas, according to the Mosaic classification. The number of deaths was evenly distributed across the time period, with 132 annual deaths (95% CI: 119 – 144) with the exception for 2020, the first year of the COVID-19 pandemic, with 183 deaths (data not shown in tables).

**Table 1:** Demographic and clinical data (*n* = 922)

Characteristics	Total (*n* = 922)
**Age,** mean years (sd)	80.2 (7.0)
18–74 years, n (%)	187 (20)
75–79 years, n (%)	224 (24)
≥80 years, n (%)	511 (56)
**NH residents,** mean years (sd)	80.6 (6.8)
**Others,** mean years (sd)	79.5 (7.3)
**Sex**	
Women, n (%)	338 (37)
Men, n (%)	584 (63)
**Mosaic** groups (SES on area level)	
Group 1 + 2 (advantaged groups), n (%)	663 (72)
Group 3 (less advantaged groups), n (%)	259 (28)
**HFRS (frailty) score**	
HFRS, not frail (group 1), n (%)	205 (22)
HFRS, frail (group 2 + 3), n (%)	717 (78)
**Specialized palliative care** (last 3 months), n (%)	120 (13)
**Nursing home residents**, n (%)	553 (60)
**ER visits last month of life** n (%)	487 (53)
**Acute hospitals as place of death** n (%)	284 (31)

### Emergency room (ER) visits during the last month of life

In total, 53% of the cohort had ER visits during the last month of life (Table 1). In univariable (unadjusted) analyses, male gender and to a greater extent frailty (HFRS), were associated with these ER visits. In the adjusted model, frailty was the only variable with an aOR of 3.90 (2.75–5.55), which was highly significant, *p* < 0.0001. In a separate model for community-dwellers (NH residents excluded), risk of frailty remained a strong variable with an aOR of 2.86 (1.61–5.07, *p* = 0.0003), whereas receipt of SPC was strongly associated with reduced need for unplanned ER visits, aOR 0.36 (0.21–0.62, *p* = 0.0002) (Table 2).

**Table 2:** Variables associated with emergency room visits in the last month of life among patients with Parkinson’s disease

Variable	Univariable analysis. *All patients*, (*n* = 922)		Multivariable analysis. *All patients*, (*n* = 922)		Multivariable analysis, *NH residents excluded,* (*n* = 369)	
	OR^a^ (95% CI)	*p*-value	aOR^b^ (95% CI)	*p*-value	aOR^b^ (95% CI)	*p*-value
Age groups						
18 – 74 years	Ref.		Ref.		Ref.	
75 – 79 years	1.06 (0.71–1.56)	0.78	1.02 (0.68–1.53)	0.94	1.42 (0.74–2.73)	0.30
≥80 years	0.74 (0.53–1.03)	0.08	0.74 (0.52–1.06)	0.10	1.59 (0.90–2.82)	0.11
Sex						
Women	Ref.		Ref.		Ref.	
Men	1.39 (1.06–1.82)	**0.02**	1.17 (0.88–1.56)	0.27	0.91 (0.56–1.49)	0.70
Socio-economic status^c^						
Mosaic groups 1 + 2	Ref.		Ref.		Ref.	
Mosaic group 3	0.92 (0.69–1.23)	0.57	0.89 (0.66–1.21)	0.46	1.23 (0.71–2.12)	0.47
HFRS^d^						
1 (not frail)	Ref.		Ref.		Ref.	
2 + 3 (frail)	3.97 (2.81–5.61)	** < 0.0001**	3.90 (2.75–5.55)	** < 0.0001**	2.86 (1.61–5.07)	**0.0003**
SPC						
No	Ref.		Ref.		Ref.	
Yes	1.11 (0.75–1.63)	0.61	0.93 (0.62–1.38)	0.72	0.36 (0.21–0.62)	**0.0002**

Univariable and multivariable analyses for all patients (*n* = 922), Multivariable analysis for patients in ordinary living (*n* = 369), Nursing home (NH) residents excluded

*Ref*  Reference group

^a^*OR *Odds ratio

^b^*aOR* adjusted Odds ratio

^c^Socio-economic status: Mosaic groups 1 + 2 are more advantaged groups, Mosaic group 3 is a less advantaged group

^d^*HFRS* Hospital Frailty Risk Score

### Receipt of specialized palliative care (SPC)

In total, 120 (13%) people received SPC during the last three months of life. However, when viewed in terms of community versus NH residency, access to SPC varied so that 21% (*n* = 79) in community-based homes and only 7% (*n* = 41) in NHs had received SPC. In univariable and multivariable analyses of the entire group (*n* = 922), people with risk of frailty (HFRS) were more than twice as likely (aOR, 2.65, 1.43–4.94, *p* = 0.002) to have received SPC that those with no frailty, (aOR, 2.65, 1.43–4.94, *p* = 0.002) in the multivariable model, Table 3). In a separate model (*n* = 369) where NH residents were excluded, being over 75 years of age was independently associated with receipt of SPC. For details see Table 3.

**Table 3:** Variables associated with receipt of specialized palliative care during the last 3 months of life among patients with Parkinson´s disease

Variable	Univariable analysis *All patients*, (*n* = 922)		Multivariable analysis *All patients*, (*n* = 922)		Multivariable analysis, *NH residents excluded,* (*n* = 369)	
	OR^a^ (95% CI)	*p*-value	aOR^b^ (95% CI)	*p*-value	aOR^b^ (95% CI)	*p*-value
Age groups						
18 – 74 years	Ref.		Ref.		Ref.	
75 – 79 years	1.56 (0.85–2.89)	0.15	1.53 (0.82–2.83)	0.17	2.39 (1.02–5.61)	**0.04**
≥80 years	1.49 (0.86–2.58)	0.15	1.54 (0.88–2.67)	0.13	3.25 (1.51–7.00)	**0.002**
Sex						
Women	Ref.		Ref.		Ref.	
Men	1.29 (0.86–1.94)	0.22	1.20 (0.79–1.83)	0.38	1.26 (0.73–2.18)	0.40
Socio-economic status^c^						
Mosaic groups 1 + 2	Ref.		Ref.		Ref.	
Mosaic group 3	0.92 (0.60–1.42)	0.71	0.94 (0.60–1.45)	0.77	1.24 (0.70–2.21)	0.46
HFRS^d^						
1 (not frail)			Ref.		Ref.	
2 + 3 (frail)	2.72 (1.46–5.04)	**0.002**	2.65 (1.42–4.94)	**0.002**	1.98 (0.89–4.41)	0.09

Univariable and multivariable analyses for all patients (*n* = 922) Multivariable analysis for patients in community-dwelling housing (*n* = 369), Nursing home (NH) residents excluded

*Ref* reference group

^a^*OR* Odds ratio

^b^*aOR* adjusted Odds ratio

^c^Socio-economic status: Mosaic groups 1 + 2 are more advantaged groups, Mosaic group 3 is a less advantaged group

^d^*HFRS* Hospital Frailty Risk Score

### Acute hospitals as place of death

Approximately one third of this patient cohort had acute hospitals as their place of death (Table 1). In the adjusted analysis, both as regards the total population (*n* = 922) and in a separate analysis of those living in the community (*n* = 369), male gender and risk of frailty were strongly associated with hospital deaths, whereas receipt of SPC was strongly associated with a low likelihood of dying in acute hospitals (Table 4). For community-dwellers, aOR for male gender was 1.90 (95% CI: 1.15–3.13, p = 0.01) and for frailty aOR was 3.70 (1.96–6.98, *p* < 0.0001), whereas aOR for receipt of SPC was 0.02 (0.01–0.07, *p* < 0.0001). For details see Table 4.

**Table 4:** Variables associated with acute hospitals as place of death, among patients with Parkinson´s disease

Variable	Univariable analysis *All patients*, (*n* = 922)		Multivariable analysis *All patients*, (*n* = 922)		Multivariable analysis, *NH residents excluded,* (*n* = 369)	
	OR^a^ (95% CI)	*p*-value	aOR^b^ (95% CI)	*p*-value	aOR^b^ (95% CI)	*p*-value
Age groups						
18 – 74 years	Ref.		Ref.		Ref.	
75 – 79 years	0.79 (0.53–1.20)	0.27	0.81 (0.52–1.25)	0.34	0.94 (0.49–1.80)	0.85
≥80 years	0.67 (0.47–0.96)	**0.03**	0.74 (0.51–1.08)	0.12	1.49 (0.83–2.65)	0.18
Sex						
Women	Ref.		Ref.		Ref.	
Men	1.61 (1.19–2.18)	**0.002**	1.54 (1.12–2.12)	**0.008**	1.90 (1.15–3.13)	**0.01**
Socio-economic status^c^						
Mosaic groups 1 + 2	Ref.		Ref.		Ref.	
Mosaic group 3	0.91 (0.66–1.24)	0.55	0.86 (0.62–1.20)	0.39	0.88 (0.51–1.53)	0.66
HFRS^d^						
1 (not frail)	Ref.		Ref.		Ref.	
2 + 3 (frail)	3.02 (1.99–4.60)	**< 0.0001**	3.44 (2.24–5.26)	**< 0.0001**	3.70 (1.96–6.99)	** < 0.0001**
SPC						
No	Ref.		Ref.		Ref.	
Yes	0.06 (0.02–0.18)	**< 0.0001**	0.05 (0.02–0.15)	**< 0.0001**	0.02 (0.01–0.07)	**< 0.0001**

Univariable and multivariable analyses for all patients (*n* = 922) Multivariable analysis for patients in community-dwelling housing (*n* = 369), Nursing home (NH) residents excluded. Multivariable analysis for patients in ordinary living (*n* = 369, of which *n* = 168 (46%) died in acute hospitals)

^a^*OR *Odds ratio

^b^*aOR *adjusted Odds ratio

^c^Socio-economic status: Mosaic groups 1 + 2 are more advantaged groups, Mosaic group 3 is a less advantaged group

^d^*HFRS *Hospital Frailty Risk Score

## Discussion

In this retrospective cohort study of 922 urban-dwellers, during the period 2015–2021, who had PD as their main diagnosis when receiving health care during their last 3 months of life, 60% were NH residents at the end-of-life. Approximately half of the entire cohort required at least one ER visit during their last month of life, and risk of frailty strongly predicted these visits. Risk of frailty was very high (78%) in the cohort and was also strongly associated with hospital deaths. Only a small minority (13% of the entire cohort) received SPC at the end of life, and this group were more likely to have a higher frailty risk. As it is, due to healthcare structural reasons, very rare that NH residents receive access to SPC, those living in ordinary housing were analyzed separately (*n* = 369). In that group, persons receiving SPC were significantly less likely to have visited an ER. In relation to place of death, men died more often in acute hospital settings, regardless of health or socioeconomic factors.

Our findings reveal that approx. half of the cohort required at least one ER visit in their last month of life − a figure in line with reports from a Canadian cohort [11]. Acute hospital admissions highlight existing gaps in the provision of quality of care among people in a palliative phase [26, 27]. People with PD who are hospitalized at the end of life undergo more invasive procedures and are more exposed to inappropriate administration of their dopaminergic regimes [20, 28]. The high proportion of ER visits in the current cohort is particularly relevant in light of evidence among patients with advanced cancer, up to one third of acute admissions are potentially avoidable [29].

Among community dwellers, the risk of frailty was the strongest predictor of unplanned ER visits. This study also adds to existing evidence concerning the very high prevalence of frailty in PD [30], when compared to cohorts dying with advanced cancer, where similar methodologies are used [31]. It should be noted that our findings recount *risk of frailty*, as HFRS is based on 109 ICD codes which can be applied retrospectively in population-based register cohorts. Nonetheless, in the clinical context, patient-reported frailty measures, such as the Frailty Phenotype [32] and Clinical Frailty Scale [33] are more frequently used and can incorporate functional and cognitive measures and clinical judgement [30]. Our exploratory findings underscore the clinical relevance of prospectively identifying those with high risk of frailty in efforts to reduce acute hospital visits at the end of life.

Thirteen percent of the current cohort received SPC at the end of life, echoing low levels of SPC receipt reported in population-based Canadian cohorts, where health care systems are also publicly funded [11, 20]. Although SPC access was higher among community dwellers (21%), this proportion is substantially lower when compared to receipt of SPC among those dying from cancer (> 75%), or chronic heart failure (24%) [34–36]. As is the case with dementia, despite the well-known progressive neurodegenerative features in PD, a PD diagnosis is not considered as terminal, which appears to act as a barrier to receiving optimal end-of-life care [37]. It is not always easy to identify when people with a PD are approaching end of life [38, 39] especially if there is a lack in specialist contact in the NH setting. The trajectory towards late-stage PD could be expected when traditional pharmacological anti-PD remedies are unfavorable with side effects and where treatment focus tends to be directed towards easement and principles of traditional palliative care. Milestones for consideration of end-of-life SPC have been propagated and include unsuitability to advanced therapy, weight loss, older age, frailty, advanced dementia and rapid deterioration in symptoms [40, 41]. Apart from enhancing the management of motor and psychological symptoms, timely referral to SPC among people with PD, could serve to meet the social and spiritual needs that are frequently overlooked in this group [15, 42–44].

Health care reimbursement structures appear to hinder NH residents, who comprise most of this cohort, from receiving SPC. Resultantly, NH dwellers with PD are susceptible to reduced access to both specialized neurological care, which compromises quality of life [45], and to specialized palliative care at the end of life. These structural inequities, not solely exclusive to the Swedish health care system [10, 21], negatively impact the quality of end-of life care, as this and other studies report an association between SPC access and a lower likelihood of acute hospitalization and hospital deaths [21]. Socioeconomic status on geographic area level did not appear to influence whether the tax financed SPC was utilized.

One third of the sample died in acute hospitals, and although reported PD hospital deaths vary widely between countries, this proportion is comparative to that (39–60%) of other European countries [46, 47]. When compared to acute hospital deaths among other disease cohorts within the Stockholm region, our findings correspond somewhat to hospital deaths among those with amyotrophic lateral sclerosis (30%)[48], chronic obstructive pulmonary disease (39%) [35], with higher levels reported for those dying due to severe heart failure (45%) [36]. Place of death is indicative of the quality of end-of life care, and acute hospitals, when compared to specialized palliative settings, are less equipped to provide optimal holistic care [49, 50]. Moreover, for older individuals, home and / or palliative care services are preferred places of death [51]. When controlled for health and socioeconomic-related factors, men with PD were more likely to die in acute hospitals settings. That men account for a larger proportion of those with PD who die in acute hospitals, has also been observed in European and North American cohorts specifically [46]. Our findings strengthen the evidence for a sex-based disparity in health care equity, which persists after controlling for health-related factors, and deserves further investigation in order to understand driving factors.

### Strengths and limitations

A main strength of this study involves use of a population-based data registry (VAL). In Stockholm, financial reimbursement to health care facilities is weighted according to VAL registry reports, and reporting is therefore mandatory. For these reasons, there is little missing data, and this registry is considered complete. This study has several limitations that deserve consideration. Ascertainment of PD diagnosis was based on the primary diagnosis during the last episode of care, as opposed to death certificate verification. It is therefore possible that cases have been omitted or misclassified due to incorrect medical journal entry at the end of life. Data concerning relevant disease-specific variables, such as PD disease severity and levodopa equivalent dosage, are not registered in the VAL database and could therefore not be accounted for in the regression analysis. We also lacked data concerning the number of, or reasons for ER visits, as well as patient preferences for end-of-life care, and although the evidence suggests that most people prefer to die in familiar residential environments as opposed to acute hospitals [52], it is possible certain participants in this cohort declined SPC. Additionally, our use of MOSAIC grouping which is a geodemographic classification system can be considered a less precise measure of socioeconomic status, when compared to other existing measures. The last two years of this study period coincide with the COVID-19 pandemic which would have affected routines regarding health care utilization. The observational nature of this study inhibits any causal associations from being inferred in relation to the outcomes in focus.

## Conclusions

We found that rates of ER visits at end of life and hospital deaths were relatively high in this population-based PD cohort. Considering a high disease burden, referral to SPC at end of life was relatively low. Sex-specific disparities in health care utilization are apparent. Our findings suggest that identifying people with high risk for frailty could assist the planning of optimal end-of-life care for people with PD.

## Data Availability

The dataset contains personally identifiable information, such as personal identity numbers, and date of death, and is therefore subject to ethical and legal restrictions on public sharing, according to Swedish laws. However, datasets generated, used and analyzed during the current study are available from the corresponding author on reasonable request.

## Abbreviations

ER: Emergency room
HFRS: Hospital Frailty Risk Score
NH: Nursing home
PD: Parkinson’s disease
SPC: Specialized palliative care
